# Dyskeratosis Congenita with Pigmentary Mosaicism and Hematopoietic Trisomy 9 in a Female Associated with a *de novo DKC1* Variant and Markedly Skewed X Chromosome Inactivation

**DOI:** 10.1038/s41525-026-00587-8

**Published:** 2026-06-09

**Authors:** Benilde García-de-Teresa, Tianna Zhao, Consuelo Salas-Labadía, Moisés Fiesco-Roa, Weiyin Zhou, Silvia Sánchez, Ulises Juárez-Figueroa, Bertha Molina, Leda Torres, Raymond Caylor, Difei Wang, Chad A. Highfill, Maryam Rafati, Jia Liu, Logan P. Zeigler, Angélica Monsivais, Esther Lieberman, María de la Luz Orozco-Covarrubias, Mauricio Rojas, Burak Altintas, Neelam Giri, Kristine M. Jones, Komal Jain, Lisa J. McReynolds, Belynda Hicks, Sara Frías, Sharon A. Savage

**Affiliations:** 1 Laboratorio de Citogenética, Instituto Nacional de Pediatría (INP), Mexico City, Mexico; 2 Cancer Genomics Research Laboratory, Frederick National Laboratory for Cancer Research, Rockville, MD USA; 3 Division of Cancer Epidemiology and Genetics, National Cancer Institute, NIH, Bethesda, MD USA; 4 Laboratorio de Genética y Cáncer, INP, Mexico City, Mexico; 5 Greenwood Genetic Center, Greenwood, SC USA; 6 Clinical Genetics Branch, DCEG, NCI, National Institutes of Health, Bethesda, MD USA; 7 Servicio de Hematología, INP, Mexico City, Mexico; 8 Departamento de Genética Humana, INP, Mexico City, Mexico; 9 Servicio de Dermatología, INP, Mexico City, Mexico; 10 Servicio de Patología, INP, Mexico City, Mexico; 11 Departamento de Medicina Genómica y Toxicología Ambiental, Instituto de Investigaciones Biomédicas, Universidad Nacional Autónoma de México, Ciudad Universitaria, Mexico City, Mexico

**Keywords:** Cell biology, Diseases, Genetics, Molecular biology

## Abstract

Pathogenic germline variants (PGVs) in dyskerin pseudouridine synthase 1 (*DKC1*) cause X-linked recessive dyskeratosis congenita (DC), a telomere biology disorder. Females with heterozygous *DKC1* PGVs rarely exhibit DC phenotypes due to favorable X chromosome inactivation (XCI). We report a female with classic DC, pigmentary mosaicism and bone marrow failure associated with a novel *de* *novo* *DKC1* variant (c.190 G > C, p.Val64Leu) with reduced expression of DKC1 and *TERC* along with downregulated signatures associated with aberrant telomere biology and ribosome function. Markedly skewed XCI was detected with expression of the mutated allele in skin fibroblasts and wild-type *DKC1* expression in the bone marrow. We hypothesize that selective pressure in the bone marrow favored wild type expressing cells which acquired a trisomy 9 but were unable to resume normal hematopoiesis. This study demonstrates *DKC1* c. 190 G > C as a likely PGV causing classic DC and highlights the molecular and clinical complexities associated with skewed XCI.

## Introduction

Dyskeratosis congenita (DC) is a rare inherited telomere biology disorder (TBD) characterized by the mucocutaneous triad of abnormal skin pigmentation, nail dystrophy and oral leukoplakia as well as very high rates of bone marrow failure^1–3^. Patients with DC have very short telomeres for their age due to pathogenic germline variants (PGVs) in genes essential for telomere maintenance and function. PGVs in *DKC1* (HGNC:2890, MIM#300126, Xq28) encoding dyskerin pseudouridine synthase 1, a component of the telomerase holoenzyme complex that extends telomere repeats, cause X-linked recessive DC (MIM#305000) and very short telomeres^4,5^.

Females with heterozygous *DKC1* PGVs usually do not develop typical clinical features due to the selective advantage of cells expressing the wild-type allele following random X-chromosome inactivation (XCI). However, some females have been reported to exhibit variably penetrant cutaneous or mild hematologic phenotypes due to skewed XCI^6–9^. Normally, XCI is a stochastic event occurring early in embryo development, resulting in random inactivation of either X chromosome in each cell^10^. This process typically creates a mixed population of cells, with roughly half containing an active paternal X chromosome and the other half containing an active maternal X chromosome^11^. Skewed XCI is defined as a ratio greater than 80:20, occurs in about 8% of females, and can result in the expression of X-linked recessive disorders in females^12^.

Herein, we report a female with classic DC due to a heterozygous novel *DKC1* variant and unfavorable highly skewed XCI. Genomic, cytogenetic and functional studies were performed to understand the underlying mechanism and remarkable complexity of her genome and phenotype.

## Results

### Clinical characterization

**Fig. 1:**
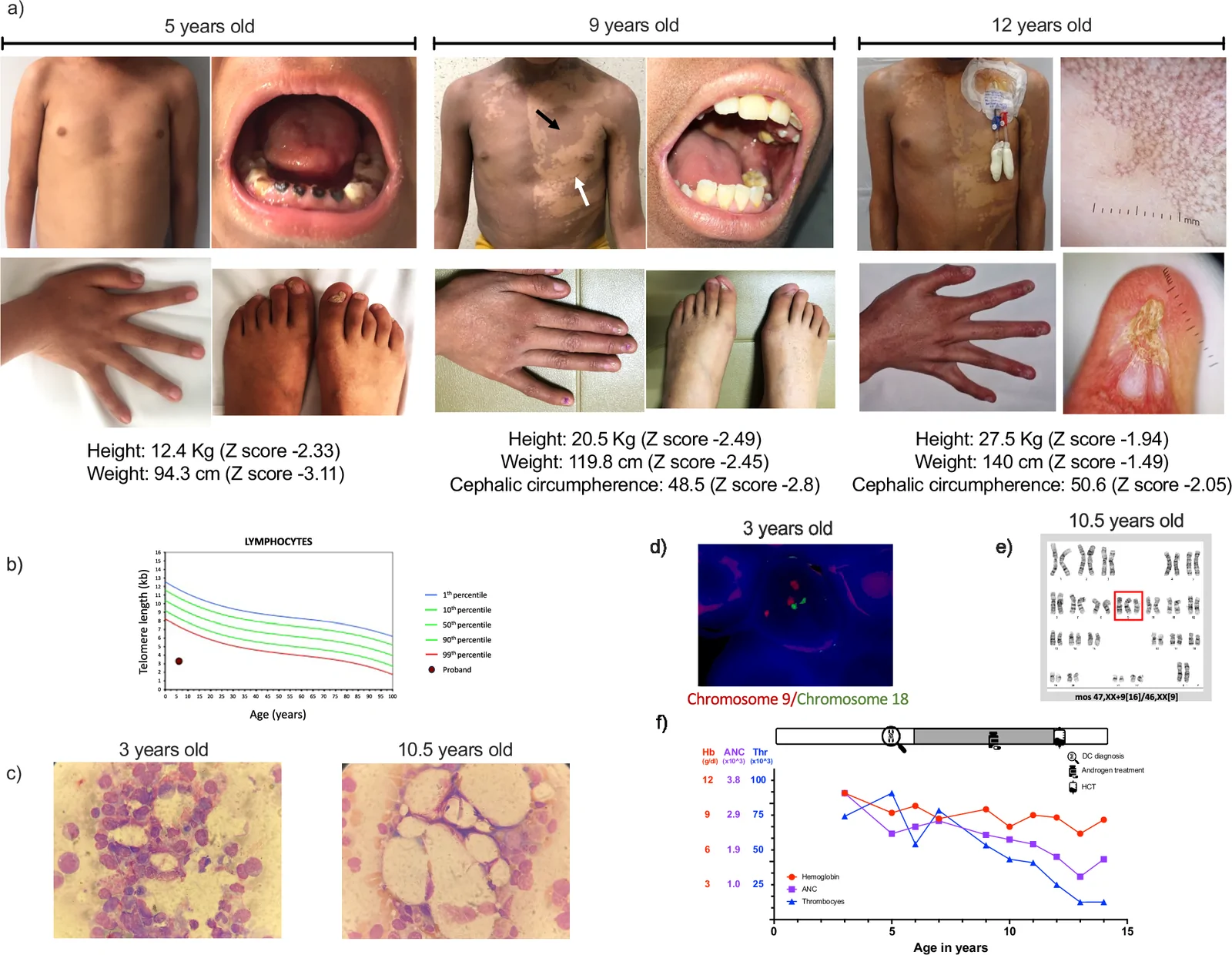
Clinical characteristics of the study participant. **a** At age 5 y (left panel) discrete pigmentary features in the upper torso and nail dystrophy of the thumb and toes were present. At age 9 y (center panel), there was evidence of pigmentary mosaicism consisting of reticulated hyperkeratotic hyperpigmentation that mainly respects the middle line, following a blaschkolinear and blocklike pattern. Black and white arrows indicate dark skin and light skin respectively. There was mild dystrophy of all fingernails and progressive dystrophy of toenails. At age 12 y (right panel) the pigmentary mosaicism appeared stable, in contrast the nail dystrophy had significantly progressed. Dermatoscopic evaluation (far right upper panel) shows a reticular pattern of pigmentation. **b** Flow FISH reveals short telomere length in total lymphocytes. **c** Representative images of 100x bone marrow aspiration smears. At the age of 3 y (left panel), bone marrow erythrocyte precursors showing normal cellularity. Myeloid cells exhibited arrested/delayed maturation, and megaloblastic changes. Marrow adipose tissue looked normal. At age 10.5 y (right panel), bone marrow exam revealed mild hypoplasia and increased marrow adipose tissue. **d** Representative FISH image showing chromosome 9 trisomy from a bone marrow smear sample obtained at age 3. **e** Karyotype of peripheral blood showing trisomy 9 in 16 of 25 analyzed metaphases at age 10.5 y. **f** Progression of cytopenias with age. The period of androgen use is shown. Hb, Hemoglobin; ANC, absolute neutrophile count; Thr, Thrombocytes; HCT, hematopoietic cell transplant.

The patient (NCI-506-1) is a 13-year-old female of Mexican ancestry who was the first child of parents whose grandmothers were distant cousins. Her mother developed preeclampsia at 28 weeks of gestation, and the patient was born via Cesarean section at 36 weeks weighing 1,950 grams. At three years (y) of age, she was hospitalized for eosinophilic colitis with protein loss and diagnosed with pancytopenia and megaloblastic anemia, with a concurrent bone marrow aspirate that showed increased cellularity and megaloblastic changes in the myeloid lineage. A diagnosis of DC was suspected at age 5 y based on the presence of irregular pigmentation affecting flexural areas and nail dystrophy (Fig. 1a). Flow cytometry with fluorescent in situ hybridization (flow FISH) revealed very short telomeres ( < 1^st^ percentile for age) in all lymphocyte subsets, supporting the diagnosis of DC (Fig. 1b, Supplementary Fig. 1a).

By nine years of age, the patient developed significant pigmentary mosaicism affecting her head, torso, and limbs. At age 12 y, dermatologic examination revealed reticulated, hyperkeratotic hyperpigmentation involving the entire body, including palms and soles. The pigmented patches showed mixed patterns of pigmentary mosaicism, with both blaschkolinear and blocklike distributions, similar to type 1b and 5 respectively as described by Kromann, et al.^13^. The skin was markedly dry with poikiloderma in sun exposed areas. Dermatoscopic examination revealed a mottled pigmentation pattern respecting follicular openings and downy hair in hypopigmented areas, while palms and soles exhibited keratoderma. The fingernails were dystrophic with splitting and longitudinal ridging (Fig. 1a). The scalp showed alopecic plaques with sparse/patches, fine hair and thick adherent scale. Intraoral examination revealed hypodontia, periodontitis, and papillary atrophy with leukoplakic patches on the left side of the tongue. Ultrasound showed normal cardiac and renal anatomy; and abdominal computerized tomography revealed a type III Michels’ anatomic variant of the hepatic artery. Brain MRI showed nonspecific focal subcortical white matter lesions but no structural abnormalities.

Bone marrow aspirates showed normal cellularity at the age of 3 y that progressed to mild hypoplasia with larger marrow adipose tissue deposits by 10.5 y of age (Fig. 1c). At the age of 3 y, chromosome 9 trisomy was identified in 30.7% of bone marrow cells (Fig. 1d). Subsequent evaluations at age 10.5 y and 12 y revealed chromosome 9 trisomy in 14 of 14 (100%) and 13 of 15 (87%) analyzed metaphases respectively, indicating a growth advantage of this clone. Cytogenetic analysis of peripheral blood at age 10.5 y showed mosaic trisomy 9 in 16 of 25 (64%) of cells (Fig. 1e). This finding was further confirmed through global screening array (GSA) mosaicism analysis performed in blood at the age of 12 y (Supplementary Fig. 1b). To determine the parental origin of the extra chromosome 9, we analyzed haplotype and allele-specific copy number in parental samples through exome sequencing and found the mosaic trisomy 9 resulted from an extra paternal copy of chromosome 9. Fibroblasts derived from dark and light skin of the patient had normal karyotypes.

With worsening pancytopenia (Fig. 1f), she began mesterolone (1 mg/kg/day) treatment at age 5 y and continued until age 12 y, achieving transfusion independence. Her hematopoietic response to mesterolone gradually declined, she lacked a matched related or unrelated donor, and thus she underwent haploidentical hematopoietic cell transplantation (HCT) from her father. Donor hematopoietic stem cells (HSCs) were prepared with ex-vivo lymphodepletion of alpha/beta T-cell receptor positive cells. The conditioning regimen consisted of fludarabine, cyclophosphamide and rabbit globulin anti-human thymocyte. The patient was discharged home 20 days after HSCs infusion. Unfortunately, after initial signs of myeloid reconstitution at 13 days post infusion, the graft failed, another HSCs infusion was not possible, and, as of publication, she remains transfusion dependent. Karyotype in bone marrow at 14.5 y showed trisomy 9 in 25 analyzed cells.

### Identification of a novel deleterious *DKC1* variant

**Fig. 2:**
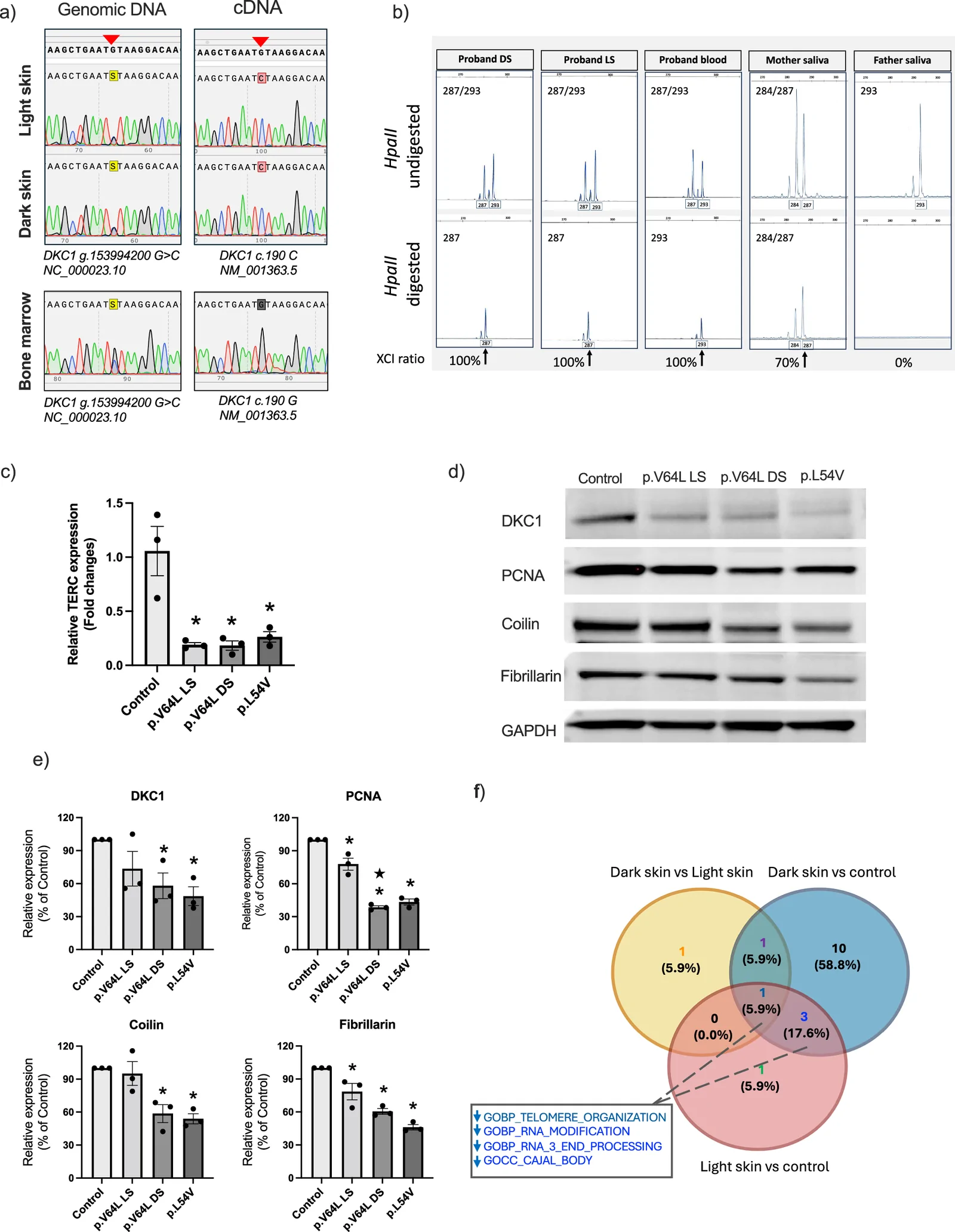
XCI and functional characterization of DKC1 p.Val64Leu. **a** Sanger sequencing electropherograms of *DKC1* variant in genomic DNA and cDNA of patient skin fibroblasts and bone marrow. **b** HUMARA XCI analysis in genomic DNA extracted from proband’s dark skin (DS), light skin (LS), blood and parental saliva of the indicated samples. Only the methylated allele (corresponding to the inactive X-chromosome) remains intact and can be amplified after HpaII digestion. **c** RT-qPCR showing the relative fold-change of *TERC* expression in the proband’s LS and DS fibroblasts and positive control (DKC1 p.Leu54Val) fibroblasts compared with wild-type control. **d** Representative western blot (WB) revealing the expression of DKC1, PCNA, coilin, and fibrillarin in skin fibroblast samples derived from an unaffected healthy individual and the patients. GAPDH was used as a loading control. **e** Quantification of WB. Data are presented as mean ( ± SEM) of protein expression relative to the wild-type control using fluorescent intensity from 3 independent WB experiments. *p < 0.05 indicates group vs control; ★*p* < 0.05 indicates p.Val64Leu LS vs p.Val64Leu DS. **f** Venn diagram showing the overlap of enriched pathways. Each lobe represents a comparison between samples and overlaps between lobes represents common enriched pathways amongst the comparisons performed. The number of pathways indicated by colors are listed in Supplementary Table 2.

A de novo heterozygous variant in *DKC1* (NM_0013663.5 :c. 190 G > C; p.Val64Leu) was identified by exome sequencing in the DNA extracted from the proband’s whole blood and skin fibroblasts derived from both dark and light skin biopsies, which was confirmed by Sanger sequencing (Fig. 2a). Neither parent carried the variant, and both maternity and paternity were genetically confirmed. No other rare variants were identified in any of the currently known DC/TBD associated genes or in other clinically relevant genes^1,14^, homozygous variants from the trio analysis are listed in supplementary Table 1. *DKC1* c.190 G > C has not been reported in the current literature, is not present in the ClinVar or gnomAD (v4.1.0) databases, and is evolutionarily conserved across 7 species (Supplementary Fig. 1c). In silico structural modelling using SWISS-MODEL^15^ shows 3D proximity of this residue to the RNA-binding PUA domain (Supplementary Fig. 1d).

### Highly skewed X-inactivation in patient’s blood and skin fibroblasts

We hypothesized that skewed XCI with complete expression of the mutant allele could be responsible for our patient’s complex DC phenotype and contribute to the pattern of mosaic skin pigmentation. At 10.5 y of age, the HUMARA assay revealed 100% skewed XCI in the patient’s blood and both light/dark skin fibroblasts (Fig. 2b). In both dark skin and light skin derived fibroblasts, the maternal allele was inactivated, whereas in blood, complete inactivation occurred in the paternal allele (Fig. 2b). Expression of the paternal allele carrying the *DKC1* variant was detected in light and dark skin fibroblasts collected at age 10.5 y. In contrast, the maternal wild type allele was expressed in the bone marrow obtained at 14.5 y (Fig. 2a). The mother’s saliva DNA had a normal random XCI pattern (70:30) (Fig. 2b). No sequence copy number variants in genes involved in XCI^16^, or other deleterious variants in genes located in chromosome X were found. G banding showed that the patient’s X chromosome structure was normal (Supplementary Fig. 1e), and sequence specific FISH confirmed that the Xq28 region was retained in the long arm of chromosome X excluding the possibility of a cryptic chromosomal rearrangement (Supplementary Fig. 1f)

### Functional characterization of DKC1 p.Val64Leu

DKC1 interacts with the telomerase RNA component, hTR (encoded by *TERC*) and plays an essential role in *TERC* stability and telomerase activity^5,17^. qPCR relative telomere length measurement showed shorter telomeres in dark and light skin derived fibroblasts compared with an age-matched control (Supplementary Fig. 1g). Compared with control fibroblasts, TERC and DKC1 expression were reduced in fibroblasts of the patient and a positive control male DC patient (*DKC1* p.Leu54Val^8^, Fig. 2c, d, e). The patient’s cells also exhibited reduced proliferation, demonstrated by the diminished expression of the cell proliferation marker PCNA similar to the p.Leu54Val sample^18^ (Fig. 2d, e), and increased cell senescence (Supplementary Fig. 1h). Given the downregulated Cajal body signatures revealed by bulk RNA sequencing (Fig. 2f), we further examined the protein expression of coilin, essential for Cajal body scaffold assembly^19^, and fibrillarin, another nucleolar protein important for ribosome biogenesis^20^. Western blot revealed reduced expression of both proteins in fibroblasts derived from the dark skin of the patient, and of only fibrillarin in light skin (Fig. 2d, e). Taken together, the p.Val64Leu variant meets likely pathogenic criteria according to the ACMG/AMP guidelines^21^.

### Dysregulated pathways and genomic instability

Given that both light and dark skin fibroblasts exclusively expressed the DKC1 p.Val64Leu variant but exhibited distinct skin pigmentation, we sought to determine whether transcriptomic changes existed. Gene set enrichment analysis (GSEA) analysis in *DKC1*-associated gene sets identified four common pathways including telomere organization, RNA modification/processing and Cajal body assembly, which were all downregulated in the patient’s light and dark skin fibroblasts compared with control fibroblasts (Fig. 2f, Supplementary Fig. 2a). Notably, in addition to shared signatures, the top 10 unique pathways identified in dark skin showed downregulation of telomerase holoenzyme, ribonucleoprotein complex, and ribosome biogenesis pathways, all of which are associated with depleted function of *DKC1* (Supplementary Table 2). Downregulation of gene subsets involved in hypopigmented skin patches was also observed in dark skin fibroblasts. Notably, the ribosome biogenesis and assembly pathways were all significantly downregulated in both light and dark skin fibroblasts, consistent with a deleterious effect caused by the defective *DKC1* variant (Supplementary Fig. 2b, Supplementary Table 3).

We further assessed the role of LINE-1 transposable element expression, which has been associated with pigmentary mosaicism^22^, in the observed mosaic skin pigmentation using bulk RNA-seq data. However, no significant difference of LINE-1 expression was found between light and dark skin fibroblasts.

We also observed no evidence of genomic instability in diepoxybutane/bleomycin treated lymphocytes (Supplementary Fig. 3a) or in scoring of non-classical chromosome aberrations in skin fibroblasts karyotypes (Supplementary Fig. 3b). Mutational signatures showed the expected spectrum (Supplementary Fig. 3c) and variant allele frequencies indicated somatic variants in the proband were similar to those in her parents (Supplementary Fig. 3d).

## Discussion

In this study, we report a female patient with a DC phenotype associated with a de novo heterozygous c. 190 G > C (p.Val64Leu) *DKC1* variant and 100% skewed XCI. To our knowledge, this is the first female *DKC1* heterozygote presenting with a classic DC phenotype^6–9^. The functional analysis revealed that the p.Val64Leu variant led to reduced expression of both *TERC* and dyskerin, consistent with the effect of other reported *DKC1* variants in male DC patients^23,24^. Notably, this variant is located in the DC-like domain (amino acids: 48-106)^25^, a known hotspot for DC-causing variants, including other N-terminal DKC1 substitutions^17,26,27^. In silico structural modelling indicates this residue lies in 3D proximity to the RNA-binding PUA domain. Therefore, DKC1 p.Val64Leu may disrupt dyskerin interactions with TERC precursors, leading to reduced *TERC* expression as observed in our patient’s fibroblasts. As a consequence of diminished *TERC* and dyskerin, affected cells may have insufficient telomerase activity and defective telomere maintenance, leading to reduced cell proliferation and increased cell senescence.

Although clonal evolution is often observed in patients with bone marrow failure, mosaic trisomy 9 has been rarely found in hematologic disorders and, to our knowledge, has not been reported in inherited bone marrow failure syndromes^28,29^. This patient had trisomy 9 in bone marrow and blood but not in fibroblasts, suggesting the mosaic trisomy 9 is an acquired event due to a mitotic error in the hematologic compartment.

**Fig. 3:**
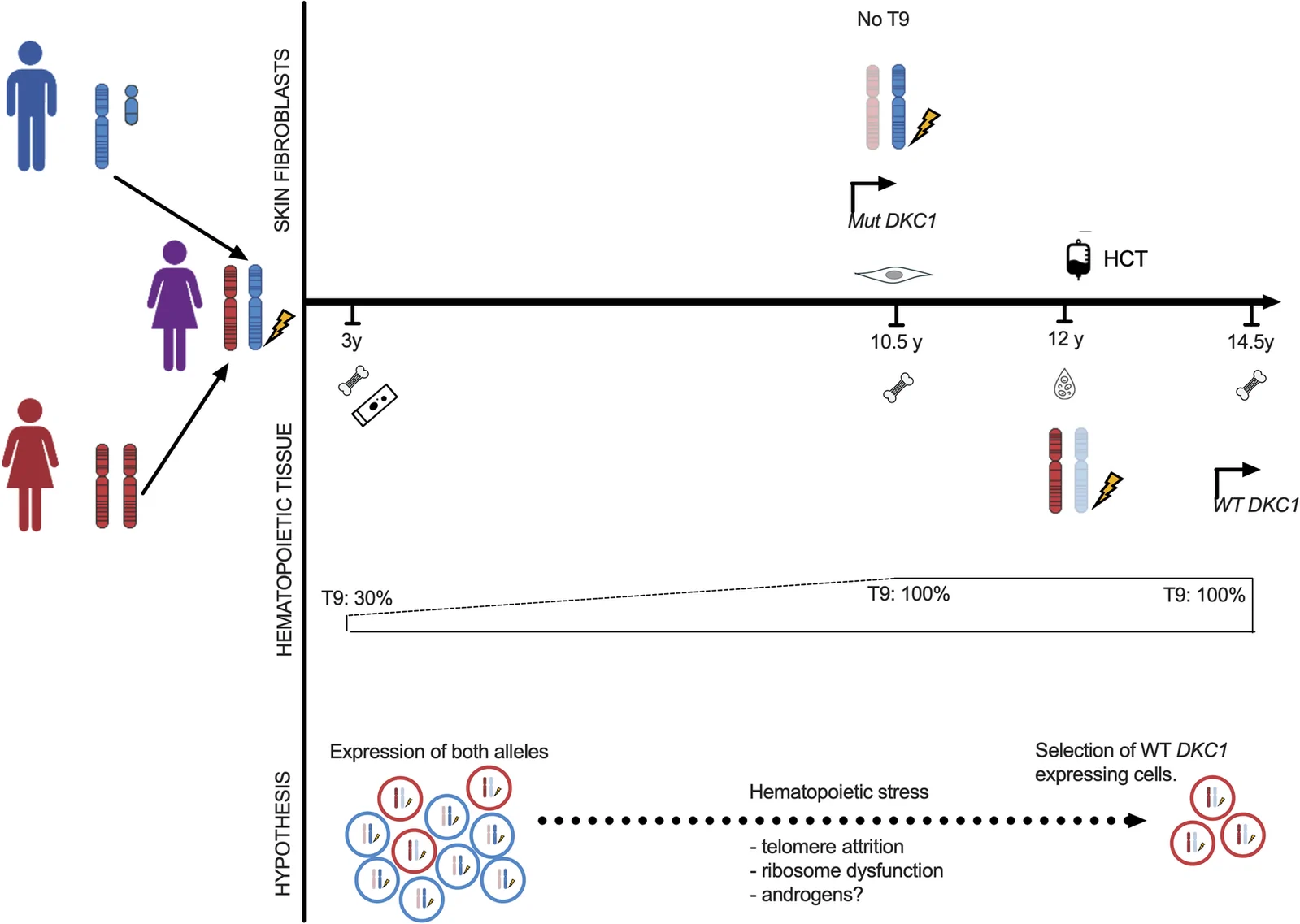
X chromosome inactivation and trisomy 9 clonal evolution timeline. The patient’s *de novo DKC1* variant c.190 G > C (denoted by yellow lightning) occurred in the paternal inherited X chromosome (blue), this may have occurred either in the germinal compartment of the mother or at early stages of development of the patient. At age 3 y, trisomy 9 was detected in 30% of bone marrow cells, by age 10.5 y, this aneuploidy affected all cells. Two years after an unsuccessful transplant cells with trisomy 9 were the only cells detected in the bone marrow. X chromosome inactivation (XCI) was studied in both dark and light skin derived fibroblasts collected at age 10 y. The maternal chromosome was inactive (light red), and mutated *DKC1* allele expression is derived from the active paternal chromosome (bright blue). The same XCI studies were performed in a blood sample collected at age 12 y, showing the opposite inactivation pattern: an active maternal chromosome (dark red) and inactive paternal chromosome (light blue). At that time, *DKC1* expression could not be assessed, but at 14.5 y, expression of *WT DKC1* was confirmed in a bone marrow sample, corresponding with the XCI pattern previously detected in blood. Note that trisomy 9 co-segregates with the *WT DKC1* expressing cells in the bone marrow. Taken together, we hypothesize that early in life, both WT and mutant *DKC1* alleles were expressed in different cells within the hematopoietic compartment. Over time, hematopoietic stress such as telomere attrition and ribosome dysfunction as well as other selective pressure, like the use of androgens, resulted in preferential survival selection of WT *DKC1* expressing cells in the bone marrow.

Most females with heterozygous *DKC1* PGVs do not develop DC features due to favorable XCI but there are a few reports of carriers with milder phenotypes including skin hyperpigmentation, nail dysplasia, early hair graying, and/or mild cytopenia (summarized in Supplementary Table 4)^6–9,30^. Notably, in our patient, highly skewed X inactivation was found on opposite X chromosomes in blood and fibroblasts, with mutant *DKC1* expression in fibroblasts at age 10.5 y and wild type *DKC1* expression in bone marrow at age 14.5 y. We hypothesize that the patient’s bone marrow expressed both alleles early in life, however, as her bone marrow failure progressed, selective pressure enabled cells expressing the maternal wild type *DKC1* allele to preferentially survive and proliferate (Fig. 3). Negative selection of *DKC1* c. 190 G > C expressing cells may not only result from telomere attrition, but also from impaired ribosome synthesis and reduced protein translation^31–35^, consistent with transcriptomic changes observed in the RNA-seq data associated with this variant. This mechanism is similar to that of abnormal ribosomal protein induced apoptosis of hematopoietic progenitors observed in Diamond–Blackfan anemia^36,37^. On the other hand, positive selection of wild type cells may have been aided by androgen-mediated clonal expansion, or even by genome topology alterations with alternative nuclear organization due to the presence of the extra chromosome 9 in these cells^38^. Unfortunately, in this patient, wild type *DKC1* expression was not enough to rescue hematopoiesis. A healthy hematopoietic niche where stromal cells can provide an appropriate microenvironment is essential for normal hematopoiesis^39^. In inherited bone marrow failure syndromes, the cells of the niche share the genetic defect which may contribute to an unfavorable microenvironment^40,41^. The *DKC1* PGV and likely unfavorable XCI in this patient’s bone marrow niche may have impaired the ability of wild type DKC1 hematopoietic cells to engraft as recently suggested^42^. This niche hypothesis would also be consistent with the patient’s failure to engraft after HCT.

The marked mosaic pattern of skin pigmentation in this patient did not resemble the skin phenotype observed in classic DC. We hypothesized a role of XCI in the patient’s marked pigmentary mosaicism^43^, but found that both dark and light skin derived fibroblasts share the same skewed XCI pattern. Single nucleotide, copy number or other genomic variants in known skin pigmentation genes did not explain pigmentary mosaicism seen in this patient. Although LINE-1 transposable elements have been associated with pigmentary mosaicism^22^, LINE-1 gene expression was similar between differently pigmented skin derived fibroblasts. GSEA analysis on bulk RNA-seq showed downregulation of gene subsets involved in hypopigmented skin patches in dark skin possibly contributing to the mosaic skin pigmentation. The mechanism underlying mosaic skin pigmentation may lie in the ectodermal derived skin cells^44^, which were not available for study in our patient.

In this patient, we excluded X chromosome structural abnormalities, PGVs in genes controlling XCI, and the presence of clinically relevant PGV of other X linked genes as potential pathogenic mechanisms for non-random XCI^45,46^. The mechanisms governing XCI, including the regulatory networks that control XIST expression and higher order chromatin organization, are incompletely understood and beyond the scope of this study^47–49^. Other limitations in this study include the resolution of exome sequencing, which does not capture genomic regions outside of coding exons that may harbor variants contributing to the phenotype; strategies such as whole genome sequencing or long-read sequencing could uncover additional variants of interest.

The combination of a *de novo DKC1* c. 190 G > C variant and 100% skewed XCI resulted in classic DC including severe bone marrow failure in a female patient. This study underscores the complexities of X-linked recessive DC associated with skewed XCI and establishes c.190 G > C as a likely pathogenic germline *DKC1* variant.

## Methods

### Study participants

The participants in this study were enrolled in the National Institutes of Health Institutional Review Board approved protocol (NCT00027274)^50^. The clinical data, photographs and biospecimens were obtained with informed consent in accordance with Health and Human Services regulation 45 CFR 46. This study was conducted at the NCI and with approval of the Institutional Review Board for Human Research and the Instituto Nacional de Pediatría (INP) in Mexico City through project 2017/040 approved by the INP’s Ethics Committee (IRB 00013674).

### Telomere length measurement

Telomere length in leukocyte subsets was measured using flow cytometry with fluorescent in situ hybridization (flow FISH) at Repeat Diagnostics, Inc (Vancouver, BC, Canada) following previously published protocols^51^. There were insufficient granulocytes for measurement. Telomere lengths in fibroblasts were quantified using quantitative PCR (qPCR) by determining the ratio of telomeric repeat copy number (T) to a single-reference gene (β-globin gene; 36B4) (S) as previously described^52^. The primers for the telomeric assay were Telo_fwd: 5’-CGGTTT(GTTTGG)5GTT-3’ and Telo_rev: 5’-GGCTTG(CCTTAC)5CCT-3’ and for the single-copy gene (36B4) assay were 36B4_fwd: 5’-CAGCAAGTGGGAAGGTGTAATCC-3’ and 36B4_rev: 5’-CCCATTCTATCATCAACGGGTACAA-3’. qPCR was performed on a LightCycler 480 (Roche). Relative T/S was calculated in relation to a standard curve, and final measurements were exponentiated to ensure normality.

### Bone marrow smear pretreatment for FISH

Before sequential dehydration in ethanol, the bone marrow smear was incubated at 37°C with red blood cell lysis solution (QIAGEN) for three hours, followed with treatment with Carnoy’s fixative (acetic acid: methanol at a ratio of 1:3) overnight at 4°C. The smear was then treated with pepsin (Porcine gastric mucosa, Sigma-Aldrich) [0.5 mg/ml] at 37 °C, for 30 s. Followed by fixing with formaldehyde/MgCl_2_.

### G-band karyotyping and FISH analysis

Peripheral whole blood, bone marrow or fibroblasts were cultured at 37 °C using the following media: 2% phytohemagglutinin supplemented RPMI medium (Gibco) for 72 h peripheral whole blood, MarrowMax (Gibco) for 48 h bone marrow, or amniomax medium (Gibco) for 2-3 days (fibroblasts). Colchicine (0.02 µg/mL for peripheral whole blood/bone marrow) or colcemid (10 mg/mL for fibroblasts) was added before harvest following standard protocols^53^. Slides were treated with 0.5% trypsin and stained with Wright–Giemsa for G-banding.

For FISH analysis, peripheral whole blood or bone marrow slides were incubated in 2×SSC at 37 °C for 30 min, followed by sequential dehydration in 70%, 85%, and 100% ethanol. Samples were co-denatured with Chromosome enumeration probes (CEP) 9 Spectrum Orange and CEP18 Spectrum Green, Abbott) at 72 °C for 2 min and hybridized at 37 °C for 48 h in a humid chamber. Slides were then washed and counterstained with DAPI/Vectashield. The FISH staining was imaged using AXIO Imager M1 or Z1 microscopes (Zeiss) with IKAROS (conventional) and ISIS (molecular) software (MetaSystems). Imaging analysis was performed according to guidelines^54^, and quantification based on 1000 nuclei for peripheral whole blood/bone marrow samples and 400 nuclei for bone marrow smears.

### Exome and Sanger sequencing

DNA extracted from the patient’s fibroblasts and blood was used for ES and SNP array genotyping, while parental saliva DNA was used for ES. ES was performed with an average depth of ∼55 X on a Novaseq 6000 sequencer (Illumina) and the reads were mapped against the GRCh37/hg19 reference genome using the Novoalign software (V4.02.01.1). Variant discovery and genotype calling were performed with GATK HaplotypeCaller (v4.0.11.0), DeepVariant (v1.0.0), and Strelka (v2.9.10).

Genomic DNA (gDNA) and cDNA from skin fibroblasts were used for *DKC1* amplification and sequencing on a Sanger sequencing platform (Applied Biosystems). The following primers are used: *DKC1* gDNA_fwd: 5’-GTATGTGGTTAAAATCGTGCATCAG-3’; *DKC1* gDNA_rev: 5’-CACAGTGCCTCCTCCCAAC-3’. *DKC1* cDNA_fwd: 5’ GCGGAAGTCATTGCCAGAAGAAG 3’; DKC1 cDNA_rev: 5’ CTGCACTCTGTTGTGACTTCACC 3’.

### X-chromosome inactivation (XCI)

XCI status was assessed using the HUMARA-PCR assay^55^ at Greenwood Genetic Center (Greenwood, SC). Genomic DNA was digested with HpaII, a methylation sensitive restriction enzyme. PCR amplification of the androgen receptor locus which contains (CAG)_n_ repeats was conducted before and after digestion, and the ratio of methylated to unmethylated alleles was measured.

### Global screening array and analysis

High-throughput, genome-wide SNP genotyping was performed using the Infinium Global Screening Array with additional custom content (v2 BeadChip for fibroblasts and v3 BeadChip for blood samples, Illumina). 200 ng of genomic DNA derived from fibroblast and blood samples were loaded as input and the genotyping was performed as described in manufacturer’s guidelines. Fluorescent labels on the BeadChips were detected using the Illumina iScan to create image files that were converted into genotype calls.

Germline Copy Number Variation (CNV) analysis was performed using PennCNV software^56^ with the GRCh37 assembly as reference, following GC wave correction of the signal intensity data. Somatic Copy Number Alteration (CNA) analysis was conducted using MoChA^57^ software with haplotypes phasing on SHAPEIT4. Both analyses were based on the GRCh37 reference genome assembly. All potential germline CNVs and somatic CNAs were plotted and reviewed, and the potential false positive calls were excluded based on the manual inspection of each plot.

### Primary fibroblast culture and western blot

Primary fibroblasts were cultured in GIBCO high glucose DMEM medium (Thermo Fisher Scientific) supplemented with 15% FBS and 1% Penicillin-Streptomycin^18^. All cells were maintained in at 37 °C and 5% CO_2_. Details of the primary skin fibroblasts are provided in Supplementary Table 5.

Western blot was conducted as previous described^58^. Protein was separated on 4–12% Bis-Tris gradient gels, then transferred onto polyvinylidene difluoride membranes (Millipore), and incubated with primary antibodies overnight at 4 °C. Protein expression was labeled with near-infrared fluorescent conjugated secondary antibodies (LI-COR), detected by Bio-Rad ChemiDoc imaging system, and quantified by Image Lab software. Statistical significance was determined by unpaired two-sided Student’s T test. Antibodies and antibody dilutions used for western blot are described in Supplementary Table 6.

### TERC quantification by RT-qPCR

Total RNA was extracted from patient’s skin (P8) and control skin fibroblasts (P12) using RNeasy Mini Kit (Qiagen) and reverse transcribed with QuantiTect Reverse Transcription Kit (QIAGEN). Real-time qPCR was conducted using Power SYBR green master mix (Applied Biosystems) on Quant Studio 7 FLEX system (Applied Biosystems). The TERC expression in each sample was normalized to housekeeping gene GAPDH. The following primers were used in this study: TERC_fwd: 5’-CTAACCCTAACTGAGAAGGGCGTA-3’, TERC_rev: 5’-GGCGAACGGGCCAGCAGCTGACATT-3’; GAPDH_fwd: 5’-GTCTCCTCTGACTTCAACAGCG-3’, GAPDH_rev: 5’-ACCACCCTGTTGCTGTAGCCAA-3’.

### β-Galactosidase senescence assay

Cellular senescence was assessed by β-galactosidase (β-gal) staining in fibroblasts derived from the proband’s light skin, proband’s dark skin, and an age/sex matched control. Cells were seeded at 0.3 × 10⁵ cells per well in triplicate in 12-well plates. After 3 days of culture, β-gal staining was performed according to the manufacturer’s instructions (Cell Signaling Technology). Cells were incubated with staining solution overnight at 37 °C in a dry incubator without CO₂. Cell nuclei were counterstained with Hoechst. Images were acquired using a Lionheart imaging system FX (Agilent Technologies), capturing nine fields per well. β-gal–positive cells were quantified using HALO image analysis software.

### Whole transcriptome sequencing and analysis

Total RNA was rRNA-depleted and reverse-transcribed, and an adapter-ligated library was prepared with the KAPA RNA HyperPrep kit with RiboErase (HMR) (Roche) using xGen Dual Index UMI Adapters (IDT) according to the manufacturer’s instructions. The paired-end sequencing was performed on an Illumina Novaseq 6000 (Illumina). For analysis, sequencing reads were merged across lanes, trimmed with Cutadapt (v2.3), and aligned to the GRCh38 reference genome using STAR (v2.7)^59^. Gene set enrichment analysis (GSEA) against C5 ontology gene sets was performed using fgsea R package^60^ (MSigDB v2023.2) and independent pathways were generated using collapsePathways function in the package. Gene sets with nominal enrichment score >1.5 or < -1.5 and Benjamini-Hochberg adjusted p-value < 0.05 were considered significant.

### Chromosome instability test

Patient peripheral blood (PB) cells were cultured in RPMI medium with 2% phytohemagglutinin and incubated with diepoxybutane (DEB) (0.1 μg/mL, Sigma Aldrich), Bleomycin (2.5 µg/mL or 5 µg/mL, Novamexan, Kemex laboratories) and vehicle control (H_2_O) for 72 h at 37 °C. A blood sample from a healthy individual was used as a negative control, with the same exposures. After incubation, colchicine [0.02 μg/mL] was added. Cells were then collected, treated with hypotonic solution and fixed using Carnoy solution (methanol 3:1 acetic acid) following standard protocol^53^. Suspension cells were spread on slides and stained with Wright and Giemsa. Twenty-five metaphases per culture were analyzed for chromosome aberrations, gaps were excluded.

### Non-classical chromosomal aberrations

The same slides used for conventional karyotyping in PB and dark and light skin fibroblasts were analyzed and scored for unclassified chromosomal abnormalities: micronucleus, nuclear buds, binucleated nuclei, bilobulated nuclei, fragmented nuclei, etc., as described^61^.

### Exome sequencing based mutational signatures in peripheral blood

Raw reads were filtered and mapped to the GRCh37/hg19 reference genome with the Burrows-Wheeler Aligner (BWA). Alignment and variant calling were performed using the Genome Analysis Toolkit (GATK), version 4.0.3. A pipeline was designed to maximize the accuracy of (somatic) variant calls with Mutect2, according to GATK Best Practices recommendations^62,63^. Variants were filtered and the vcf file was used as input to detect mutational signatures using the SigProfiler extractor tool^64,65^.

## Supplementary information

Supplementary Information

## Data Availability

There are restrictions to the availability of data from clinicaltrials.gov Identifier: NCT00027274 due to the importance of maintaining participant confidentiality. De-identified data from that study may be requested from the corresponding authors and will be made available to qualified scientists after completion of appropriate data transfer agreements and NIH Institutional Review Board approval.
