# Supplementary material for: Dyskeratosis Congenita with Pigmentary Mosaicism and Hematopoietic Trisomy 9 in a Female Associated with a de novo DKC1 Variant and Markedly Skewed X Chromosome Inactivation
Source: NPJ Genom Med. 2026 Jun 9;11:55. doi: 10.1038/s41525-026-00587-8 (PMC13594237; doi:10.1038/s41525-026-00587-8)
Supplement: Supplementary file 1 [file 41525_2026_587_MOESM1_ESM.pdf]

Supplementary Figure 1

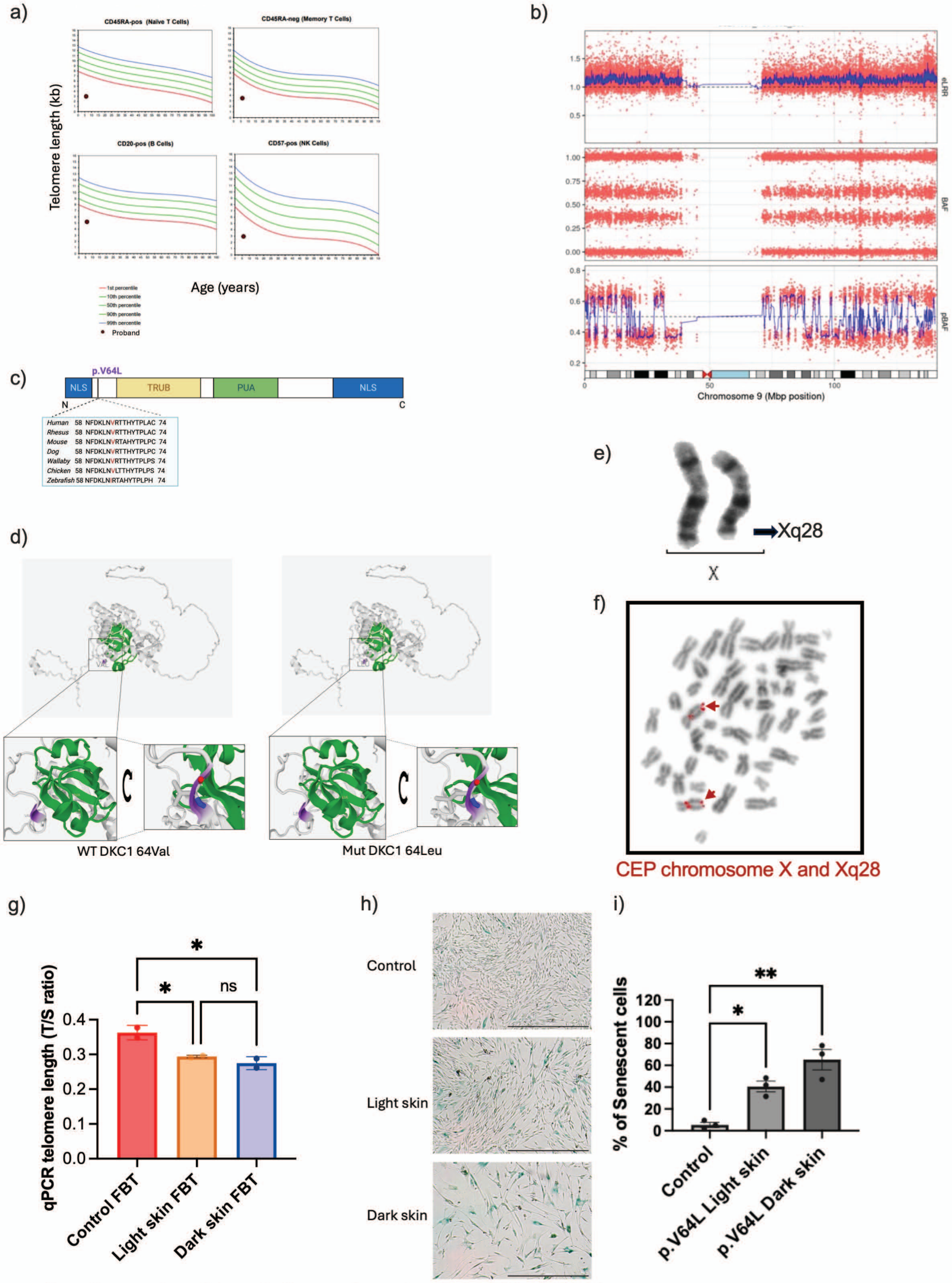

Supplementary figure 1. Genomic and cytogenetic analysis.

a) Flow FISH reveals telomere length in 4 lymphocyte subsets. The population distribution of telomere length in kilobases (kb) is represented by colored lines. **b)** Chromosome 9 copy number variation analysis of genotyping data from the patient's blood sample. eLRR: Estimated Log R Ratio, BAF: B Allele Frequency, pBAF: Phased B Allele Frequency. **c)** Schematic of DKC1 protein functional domains and variant annotation (created using BioRender). **d)** Structural modeling of dyskerin was performed using SWISS-MODEL. The wild-type (Val64) structure is shown on the left, and the 64Leu variant is shown on the right. Green indicates the PUA domain; violet highlights the amino acid at position 64; red represents the carboxyl group; and blue represents the amino group. **e)** Representative G-banding resolution image of chromosome X pair from the patient's peripheral blood. **f)** Single sequence FISH probe assay signaling in red the centromere of the X chromosome and, signaled by red arrows, the Xq28 region of both homologues. CEP: chromosome enumeration probes. **g)** qPCR relative telomere lengths of DNA from the patient's skin fibroblasts compared with control. *$p<0.05$. **h)** Representative images of cell senescence β-gal staining in proband and control fibroblasts; quantification of senescent cells is shown in **(i)** *$p<0.05$, **$p<0.01$. Scale bar: 1000 μm.

Supplementary Figure 2

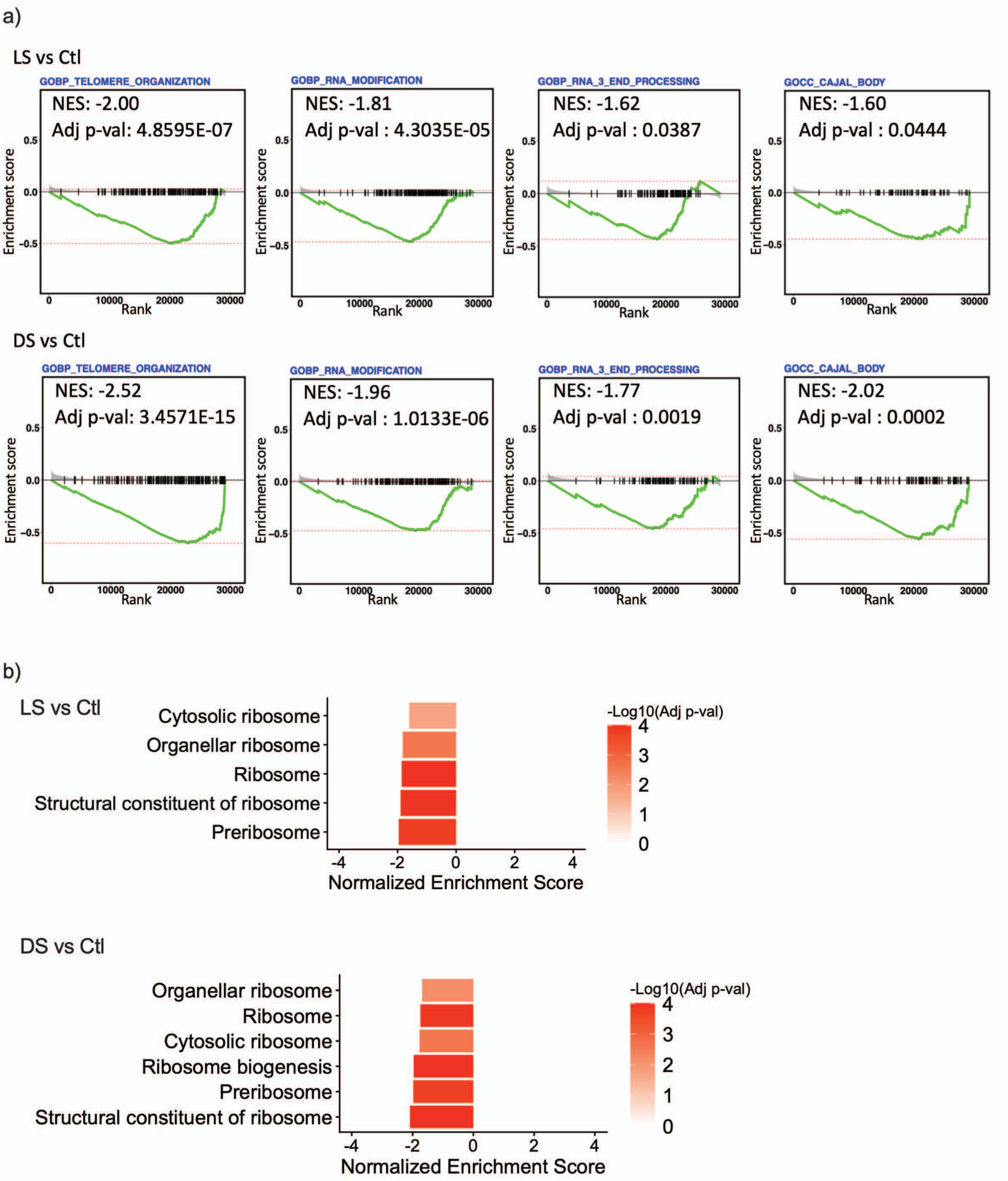

Supplementary figure 2. Gene set enrichment analysis (GSEA).
a) Enrichment plots of GSEA analysis showing significantly downregulated signatures associated with canonical DKC1 function in patient skin. Abbreviations: NES, normalized enrichment score; Adj p-val, adjusted P value. Gene sets with NES < -1.5 (or > 1.5) and Adj p-val < 0.05 are considered statistically significant. NES >1.5 indicates upregulated and NES <-1.5 indicates downregulated pathways. LS: light skin, Ctl: control, DS: dark skin. **b**) Bar plot revealing the statistically significant downregulated pathways. Gene sets with NES < -1.5 and adjusted p value ($^{Adj\ p\text{-}val}$) < 0.05 are considered statistically significant. The color scale indicates significance, shown as -Log10$^{(Adj\ p\text{-}val)}$.

Supplementary Figure 3

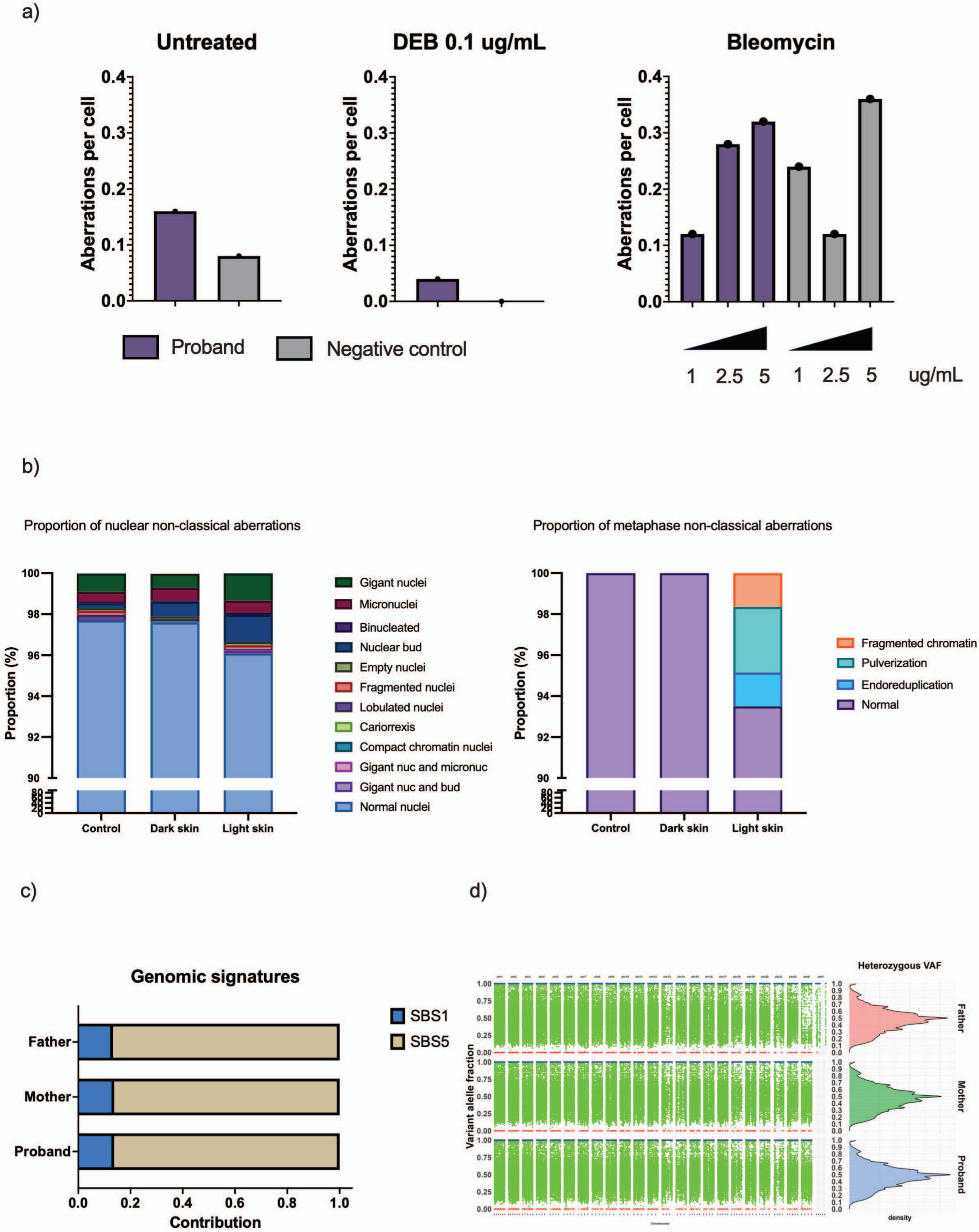

Supplementary figure 3. Evaluation of genome instability.
a) Chromosome breakage studies. Chromosomal aberrations were scored in untreated, and diepoxybutane (DEB) and bleomycin exposed lymphocytes in both proband and negative control lymphocytes. **b)** Proportion of cells with nuclei aberrations (left) or non-classical chromosomal aberrations (right) from karyotype slides. **c)** Histogram of COSMIC SBS1 and SBS5 mutational signatures contribution to the mutational spectra of the proband's PB cells and oral mucosa from her parents. **d)** Variant allele fraction profiles of the patient and her parents.

Supplementary Table 1: Homozygous variants of trio analysis with a Franklin pathogenic classification

Gene	Variation Typ	Chr	Start Positio	Stop Positio	Ref	Alt	dbSNP	Transcript	AA Change	Nucleotide	Exon	Zygosity	Region	Effect	Confidence	Quality	Genotype Qu	Genotype Lik	Depth	Ref Depth	Obs Depth
NDC1	SNP	chr1	53787222	53787222	T	C	rs775500548	NM_018087.	p.Asp579Gly	c.1736A>G	16	hom	Exonic	Missense	Low	36.07	6	49:06:00	2	0	2
NKRF	Indel	chrX	119605952	119605952	C	CG	rs5903560	NM_017544.5		c.42-1dupC	2	hom	Splice Acceptor		Low	3155.69	78	971:78:0	26	0	26
TCEAL6	Indel	chrX	102140808	102140808	T	TG	rs200987681	NM_0010069	p.Gln175fs	c.523dupC	3	hom	Exonic	Frameshift	Low	7209.69	99	2542:229:0	76	0	76
NUDT11	Indel	chrX	51496443	51496457	ATCCTCGAG	A	rs78182391	NM_018159.	p.Met1fs	c.-13_1delG(	1	hom	Exonic	Start Loss	Low	11788.7	99	3863:265:0	87	0	87
FCGBP	Indel	chr19	39906061	39906061	A	AGG	rs133058324	NM_003890.	p.Glu1234fs	c.3698_3699	15	hom	Exonic	Frameshift	Low	1696.66	60	900:60:0	20	0	20
FCGBP	Indel	chr19	39906057	39906059	CCT	C	rs132668018	NM_003890.	p.Glu1234fs	c.3701_3702	15	hom	Exonic	Frameshift	Low	1606.55	63	945:63:0	21	0	21
HLA-DRB1	Indel	chr6	32581596	32581616	CCACTTGGC	C	rs140357311	NM_002124.	p.Glu198fs	c.593_612de	3	hom	Exonic	Frameshift	Low	33.86	3	45:03:00	3	0	1
HLA-B	Indel	chr6	31356751	31356751	G	GTT	rs749557905	NM_005514.	p.Gln94fs	c.279_280ins	2	hom	Exonic	Frameshift	Low	2821.12	84	1260:84:0	30	0	28
CDCP2	Indel	chr1	54139646	54139646	G	GC	rs36013100	NM_201546.	p.Met409fs	c.1223_1224	4	hom	Exonic	Frameshift	Low	11090.9	99	3422:264:0	88	0	88
OR2T7	Indel	chr1	248441258	248441258	T	TTC	rs58223479	NM_0013860	p.Phe19fs	c.54_55dup(	2	hom	Exonic	Frameshift	Low	2620.69	51	739:51:00	17	0	17
RBM48	SNP	chr7	92534717	92534717	C	T	rs191557887	NM_032120.	p.Ser255Leu	c.764C>T	4	hom	Exonic	Missense	High	2764.98	99	1224:99:0	33	0	33

Gene	Aggregated F	1000 genome	ESP 6500	ExAC (All)	gnomAD (Exo	gnomAD (Ge	UK10K Twins	UK10K ALSPA	Internal Sam	Internal Sam	Aggregated P	SIFT Pred	SIFT Score	POLYPHEN2	POLYPHEN2	MUT TASTER	MUT TASTER	MUT ASSESS	MUT ASSESS	FATHMM Pre	FATHMM Sco	GERP	REVEL Pred	REVEL Score
NDC1	8.33E-06	N/A	N/A	8.33E-06	N/A	N/A	N/A	N/A	1 (0.93%)	[]	0.98398876	Deleterious	0			D	1	Med	2.485	Uncertain	-3.23	4.44	Deleterious	0.943
NKRF	3.00E-05	N/A	N/A	N/A	N/A	0.99970615	N/A	N/A	41 (37.96%)	[]														
TCEAL6	3.00E-05	N/A	0.97682762	0.99264258	N/A	0.99952161	N/A	N/A	51 (47.22%)	[]	0.01													
NUDT11	3.00E-05	N/A	0.92813337	0.46304318	N/A	0.99710465	N/A	N/A	76 (70.37%)	[]														
FCGBP	3.00E-05	N/A	N/A	N/A	N/A	0.99995554	N/A	N/A	17 (15.74%)	[]														
FCGBP	3.00E-05	N/A	N/A	N/A	N/A	0.973607	N/A	N/A	18 (16.67%)	[]	0.01													
HLA-DRB1	3.00E-05	N/A	N/A	N/A	N/A	0.00797846	N/A	N/A	1 (0.93%)	[]														
HLA-B	3.00E-05	N/A	N/A	0.70535362	N/A	0.4341636	N/A	N/A	6 (5.56%)	[]														
CDCP2	3.00E-05	N/A	N/A	0.69836456	N/A	0.69325352	N/A	N/A	66 (61.11%)	[]														
OR2T7	3.00E-05	N/A	N/A	0.00070749	N/A	0.85707271	N/A	N/A	7 (6.48%)	[]														
RBM48	3.35E-05	N/A	N/A	0.00016578	N/A	3.29E-05	N/A	N/A	4 (3.70%)	[]	0.30970497	Uncertain	0.011			N	1.30E-05	Med	2.645			4.42	Benign	0.187

Gene	Omim	Omim Gene	ClinVar	ClinVar Clas	GWAS	User Classifi	Include In Re	Clinically Irre	Clinical Inter	Genoox Classification	Father - Zygosity	Father - Ref Depth	Father - Alt Depth	Mother - Zygosity	Mother - Ref Depth	Mother - Alt Depth	Inheritance Model
NDC1							False	False		Likely Pathogenic	N/A	N/A	N/A	N/A	N/A	N/A	De Novo
NKRF							False	False		Uncertain - Possibly Pathogenic (Moderate)	hom	0	23	hom	0	36	N/A
TCEAL6							False	False		Uncertain - Possibly Pathogenic (Moderate)	hom	0	44	hom	0	95	N/A
NUDT11							False	False		Uncertain - Possibly Pathogenic (Moderate)	hom	0	62	hom	0	117	N/A
FCGBP							False	False		Uncertain - Possibly Pathogenic (Moderate)	hom	0	9	hom	0	9	N/A
FCGBP							False	False		Uncertain - Possibly Pathogenic (Moderate)	hom	0	6	hom	0	9	N/A
HLA-DRB1							False	False		Uncertain - Possibly Pathogenic (Moderate)	N/A	N/A	N/A	N/A	N/A	N/A	De Novo
HLA-B							False	False		Uncertain - Possibly Pathogenic (Moderate)	hom	0	32	hom	0	3	N/A
CDCP2							False	False		Uncertain - Possibly Pathogenic (Moderate)	het	55	97	hom	3	146	N/A
OR2T7							False	False		Uncertain - Possibly Pathogenic (Low)	hom	0	23	hom	0	21	N/A
RBM48			http://www.ncbi.nlm.nih.gov/clinvar/RCV001941464				False	False		Uncertain - Possibly Pathogenic (Low)	het	18	33	het	21	22	Autosomal Recessive

Export Summary:
Date of Expor 2/23/26

Filters Applied:
Zygosity Homozygote
Franklin Clas Pathogenic
Filter logic scv

Supplementary Table 2: Significantly Enriched Signatures Across Comparisons

	Enriched pathways	Signatures	NES	padj	Regulation	No. of leading edge genes
Dark skin vs Control	1*	GOBP_TELOMERE_ORGANIZATION	-2.52	3.46E-15	Down	182
Light skin vs Control			-2	4.86E-07		
Dark skin vs Light skin			-2.02	3.43E-07		
Dark skin vs Control	3	GOBP_RNA_3_END_PROCESSING	-1.77	0.001935	Down	92
Light skin vs Control			-1.62	0.03865		
Dark skin vs Control		GOBP_RNA_MODIFICATION	-1.96	1.01E-06	Down	173
Light skin vs Control			-1.81	4.30E-05		
Dark skin vs Control		GOCC_CAJAL_BODY	-2.02	0.0001999	Down	73
Light skin vs Control			-1.6	0.04435		
Light Skin vs Control	1	GOBP_PROTEIN_LOCALIZATION_TO_CHROMOSOME	-1.87	1.78E-06	Down	113
Dark skin vs Light Skin	1	GOBP_REGULATORY_NCRNA_MEDIATED_GENE_SILENCING	-1.86	1.05E-09	Down	318
Dark skin vs Control	1	GOBP_REGULATION_OF_CHROMOSOME_ORGANIZATION	-2.46	3.70E-17	Down	240
Dark skin vs Light Skin			-2.16	1.02E-13		
Dark skin vs Control	10	GOBP_RNA_LOCALIZATION	-1.52	0.005221	Down	188
		HP_TELANGIECTASIA_OF_THE_SKIN	-1.59	0.0278	Down	72
		HP_LEUKEMIA	-1.66	0.003756	Down	152
		GOCC_SNO_S_RNA_CONTAINING_RIBONUCLEOPROTEIN_COMPLEX	-1.75	0.04663	Down	25
		GOCC_TELOMERASE_HOLOENZYME_COMPLEX	-1.77	0.04466	Down	21
		HP_HYPOPIGMENTED_SKIN_PATCHES	-1.78	0.002104	Down	108
		GOBP_RRNA_METABOLIC_PROCESS	-1.89	4.45E-08	Down	257
		GOBP_RIBOSOME_BIOGENESIS	-1.92	6.03E-11	Down	316
		HP_MYELODYSPLASIA	-1.93	0.0002367	Down	110
		GOMF_CATALYTIC_ACTIVITY_ACTING_ON_DNA	-2.03	3.81E-09	Down	239

*The colored numbers correspond to their location in Venn diagram of Figure 2E.

Supplementary Table 3: Significantly Enriched Ribosome Signatures

**Light skin vs Control**	padj	NES	Regulation	Size
Ribosome	6.09E-07	-1.8633417	Down	225
Structural constituent of ribosome	1.40E-05	-1.9001351	Down	158
Preribosome	5.31E-05	-1.9639226	Down	106
Organellar ribosome	0.00225068	-1.8265444	Down	88
Cytosolic ribosome	0.01791968	-1.6061963	Down	112

**Dark skin vs Control**	padj	NES	Regulation	Size
Ribosome biogenesis	7.85E-10	-1.9658106	Down	316
Structural constituent of ribosome	1.14E-07	-2.0903559	Down	158
Ribosome	2.18E-05	-1.7462393	Down	225
Preribosome	7.85E-05	-1.9783879	Down	106
Cytosolic ribosome	0.00222204	-1.7724476	Down	112
Organellar ribosome	0.00687894	-1.6884875	Down	88

Supplementary Table 4: Summary of Reported Symptomatic Female *DKC1* Heterozygotes

Case	Author	Year of publication	PMID	ID	Familial or de novo	*DKC1 variant*	Symptoms: Hematologic	Liver	Skin hyperpigmentation	Nail dysplasia	Teeth	Early hair graying	Early hair loss	Eye	Wound healing	Other
1	K Devriendt, et al	1997	9042917	II-2	familial	unknown				brittle nails						
2				II-10	familial	unknown			typical of DC	typical of DC						
3				III-4	familial	unknown			typical of DC, later Blaschko lines			yes	yes			
4	JK Alder, et al	2013	23946118	Fam 1 II-1	familial	c.145A>T, p.Thr49Ser						yes			dehiscence	
5				Fam 2 I-2	familial	c.1226C>G, p.Pro409Arg			typical of DC	typical of DC						
6				Fam 2 III-2	familial	c.1226C>G, p.Pro409Arg			typical of DC	typical of DC				retinal macroaneurism	delayed	anosmia
7				Fam 2 III-5	familial	c.1226C>G, p.Pro409Arg			typical of DC		fragile, caries					cutaneous telangiectasia
8				Fam 2 III-7	familial	c.1226C>G, p.Pro409Arg			typical of DC	typical of DC		yes				developmental delay
9	J Xu, et al	2016	27570172	NCI 288-3	familial	p.L54V	Aplastic anemia	Non alcoholic cirrhosis		abnormal nails	early tooth loss					
10				NCI 288-4	familial	p.L54V				ridged nails			alopecia			
11				NCI-219-2	familial	p.deltaE35			skin hyperpigmentation	nail dystrophy		yes				
12	EAM Hirvonen, et al	2019	30185935	Mother	familial	c.1218_1219insCAG p.Asp406_Ser407insGln	No		no	no	no	yes				
13				Aunt 1	familial	c.1218_1219insCAG p.Asp406_Ser407insGln	Mild anemia (Hb 11.4)		yes	yes	aggresive periodontitis	yes				
14				Aunt 2	familial	c.1218_1219insCAG p.Asp406_Ser407insGln	Mild anemia (Hb 10.9) Mild lymphopenia (ANC 1.3)		yes	yes	no	yes				
15	Present case			NCI 506-1	*de novo*	c.190G>C p.V64L	BMF, Pancytopenia		pigmentary mosaicism	yes	severe tooth decay		yes	dacriostenosis		

Case	X chromosome inactivation studies and telomere length: Hematologic compartment: XCI ratio	Hematologic compartment: Expressed allele	Hematologic compartment: Telomere length	Buccal mucosa: XCI ratio	Buccal mucosa: Expressed allele	Tongue: XCI ratio	Tongue: Expressed allele	Light skin fibroblasts: XCI ratio	Light skin fibroblasts: Expressed allele	Light skin fibroblasts: Telomere length	Dark skin fibroblasts: XCI ratio	Dark skin fibroblasts: Expressed allele	Dark skin fibroblasts: Telomere length
1	>95:5												
2	>95:5	paternal											
3	>95:5			Random				>95:5	paternal		>95:5	paternal	
4	93%	not studied	5th percentile										
5													
6	100%	not studied											
7	100%	not studied											
8													
9			normal										
10	skewed	WT (paternal) EBV immortalized lymphocyte cell line	normal										
11	skewed	WT (paternal) EBV immortalized lymphocyte cell line	normal	skewed				skewed			skewed		
12		WT 100% (paternal)			WT 63% (paternal)		WT 86% (paternal)						
13		WT 55% (paternal)			WT 55% (paternal)		WT 72% (paternal)						
14		WT 95% (paternal)			WT 93% (paternal)		WT 92% (paternal)						
15	100:0	WT maternal	< 1st percentile					100:0	DKC1 mut paternal	reduced	100:0	DKC1 mut paternal	reduced

Abbreviations: BMF, bone marrow failure; DC, dyskeratosis congenita; Hb, hemoglobin; WT, wild-type; XCI, X-chromosome inactivation

Supplementary Table 5: Information on primary fibroblasts used in this study

Cell samples	DKC1 mutation	Sex	Age of sampling (years)
Control	none	Female	9
Light skin	p.V64L	Female	9
Dark skin	p.V64L	Female	9
Positive control	p.L54V	Male	46

Supplementary Table 6: Antibodies and Antibody Dilutions

Primary Antibodies	Supplier	Catalogue #	Dilution
DKC1	Santa Cruz	sc-373956	1/500
PCNA	Cell Signaling	13110T	1/1000
PARP	Cell Signaling	9542T	1/1000
Coilin	Cell Signaling	14168	1/1000
Fibrillarin	Cell Signaling	2639T	1/1000
GAPDH	Cell Signaling	97166T	1/1000
**Secondary Antibodies**	**Supplier**	**Catalogue #**	**Dilution**
IRDye® 800CW Goat anti-Rabbit IgG	LI-COR	926-32211	Western blot (1/10,000)
IRDye® 680RD Goat anti-Mouse IgG	LI-COR	925-68070	Western blot (1/10,000)

Western blot images

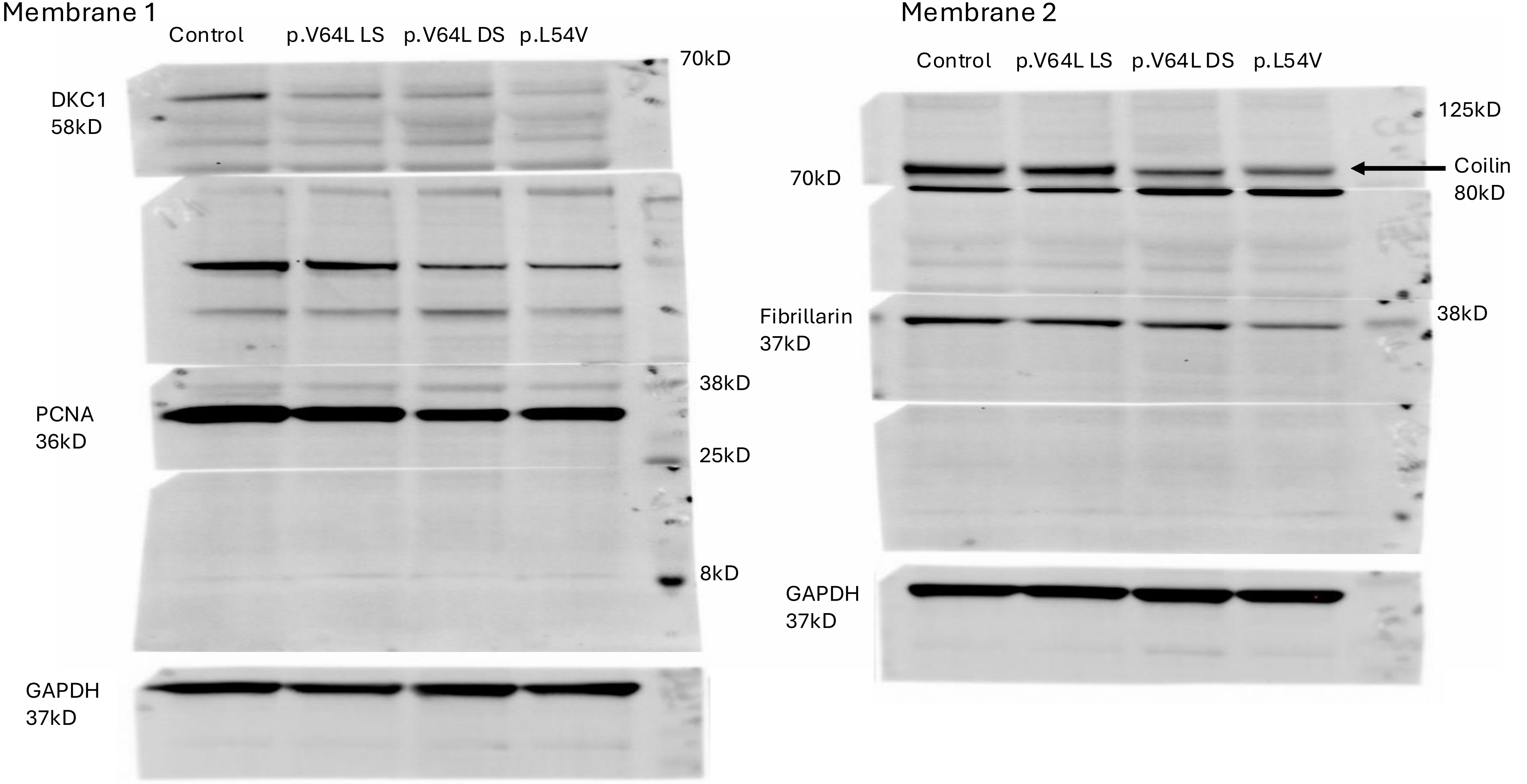

Figure legend: Given the limited amount of protein sample and to reduce antibody consumption, membranes were cropped into smaller pieces according to molecular weight and incubated with specific antibodies for each target. The cropped membrane sections were subsequently assembled andimaged together using the Bio-Rad ChemiDoc imaging system.

To detect the expression of PCNA, fibrillarin, and GAPDH which have similar molecular weights (~37kD), antibodies raised in different species and labeled with spectrally distinct fluorophores were used to avoid cross-reactivity. PCNA and fibrillarin were probed on two different membranes (1 and 2) using rabbit primary antibodies and detected with LI-COR IRDye 800CW (green) secondary antibody, followed by imaging using a Bio-Rad ChemiDoc system. GAPDH was subsequently incubated with a mouse primary antibody and detected by LI-COR IRDye 680RD (red) secondary antibody.

Membrane 1

Raw image for DKC1, PCNA and coilin and Fibrillarin blot

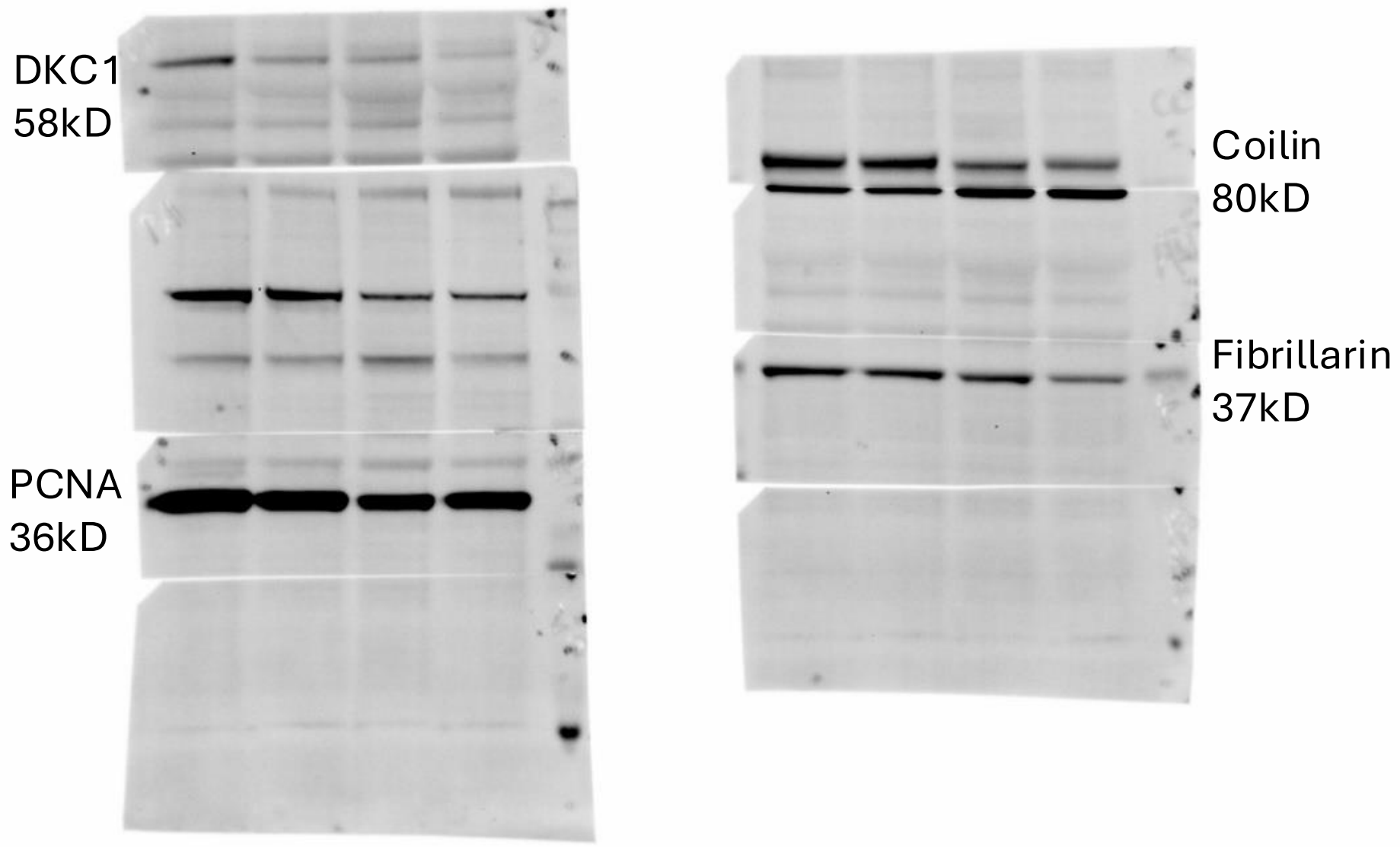

Membrane 2

Raw image for GAPDH blot

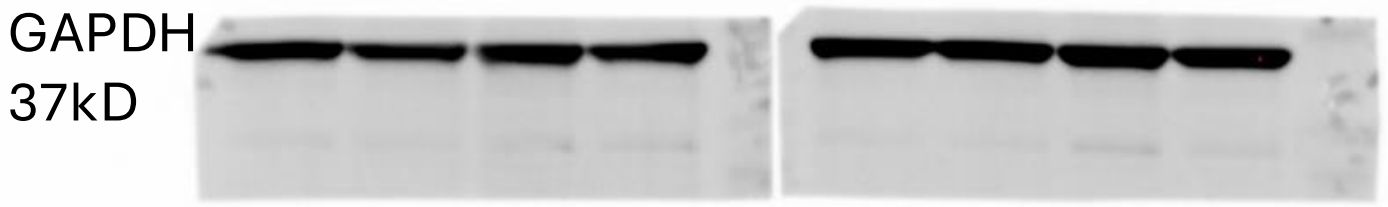

CARE case report guidelines

CARE Checklist of information to include when writing a case report

(cc) BY-NC-ND

Topic	Item	Checklist item description	Reported on Line
**Title**	1	The diagnosis or intervention of primary focus followed by the words "case report"	1-2
**Key Words**	2	2 to 5 key words that identify diagnoses or interventions in this case report, including "case report"	35
**Abstract (no references)**	3a	Introduction: What is unique about this case and what does it add to the scientific literature?	38-44
	3b	Main symptoms and/or important clinical findings	42-43
	3c	The main diagnoses, therapeutic interventions, and outcomes	41-45
	3d	Conclusion—What is the main "take-away" lesson(s) from this case?	47-49
**Introduction**	4	One or two paragraphs summarizing why this case is unique (**may include references**)	51-70
**Patient Information**	5a	De-identified patient specific information.	73
	5b	Primary concerns and symptoms of the patient.	73-119
	5c	Medical, family, and psycho-social history including relevant genetic information	73-119, 121-146
	5d	Relevant past interventions with outcomes	109-119
**Clinical Findings**	6	Describe significant physical examination (PE) and important clinical findings.	Fig 1
**Timeline**	7	Historical and current information from this episode of care organized as a timeline	Fig 1 and Fig 3
**Diagnostic Assessment**	8a	Diagnostic testing (such as PE, laboratory testing, imaging, surveys).	73-119, 121-146
	8b	Diagnostic challenges (such as access to testing, financial, or cultural)	NA
	8c	Diagnosis (including other diagnoses considered)	120-185
	8d	Prognosis (such as staging in oncology) where applicable	109-119
**Therapeutic Intervention**	9a	Types of therapeutic intervention (such as pharmacologic, surgical, preventive, self-care)	NA
	9b	Administration of therapeutic intervention (such as dosage, strength, duration)	NA
	9c	Changes in therapeutic intervention (with rationale)	NA
**Follow-up and Outcomes**	10a	Clinician and patient-assessed outcomes (if available)	NA
	10b	Important follow-up diagnostic and other test results	NA
	10c	Intervention adherence and tolerability (How was this assessed?)	NA
	10d	Adverse and unanticipated events	NA
**Discussion**	11a	A scientific discussion of the strengths AND limitations associated with this case report	241-249
	11b	Discussion of the relevant medical literature **with references**.	188-249
	11c	The scientific rationale for any conclusions (including assessment of possible causes)	239-261
	11d	The primary "take-away" lessons of this case report (without references) in a one paragraph conclusion	250-253
**Patient Perspective**	12	The patient should share their perspective in one to two paragraphs on the treatment(s) they received	NA
**Informed Consent**	13	Did the patient give informed consent? Please provide if requested	**Yes** ☒ **No** ☐
